# Identification of potential biomarkers and immune infiltration characteristics in recurrent implantation failure using bioinformatics analysis

**DOI:** 10.3389/fimmu.2023.992765

**Published:** 2023-01-26

**Authors:** Zhen-Zhen Lai, Jie Zhang, Wen-Jie Zhou, Jia-Wei Shi, Hui-Li Yang, Shao-Liang Yang, Jiang-Nan Wu, Feng Xie, Tao Zhang, Ming-Qing Li

**Affiliations:** ^1^ Laboratory for Reproductive Immunology, Hospital of Obstetrics and Gynecology, Fudan University, Shanghai, China; ^2^ NHC Key Lab of Reproduction Regulation, Shanghai Institute for Biomedical and Pharmaceutical Technologies, Fudan University, Shanghai, China; ^3^ The International Peace Maternity and Child Health Hospital, School of Medicine, Shanghai Jiao Tong University, Shanghai, China; ^4^ Center of Reproductive Medicine of Ruijin Hospital, School of Medicine, Shanghai Jiao Tong University, Shanghai, China; ^5^ Clinical Epidemiology, Hospital of Obstetrics and Gynecology, Shanghai Medical School, Fudan University, Shanghai, China; ^6^ Center for Diagnosis and Treatment of Cervical and Uterine Diseases, Hospital of Obstetrics and Gynecology, Fudan University, Shanghai, China; ^7^ Assisted Reproductive Technology Unit, Department of Obstetrics and Gynecology, Faculty of Medicine, Chinese University of Hong Kong, Hong Kong, Hong Kong SAR, China; ^8^ Shanghai Key Laboratory of Female Reproductive Endocrine Related Diseases, Hospital of Obstetrics and Gynecology, Fudan University, Shanghai, China

**Keywords:** recurrent implantation failure, immune infiltration, Wnt/β-catenin signaling, notch signaling, biomarkers, bioinformatics

## Abstract

**Introduction:**

Recurrent implantation failure (RIF) is a frustrating challenge because the cause is unknown. The current study aims to identify differentially expressed genes (DEGs) in the endometrium on the basis of immune cell infiltration characteristics between RIF patients and healthy controls, as well as to investigate potential prognostic markers in RIF.

**Methods:**

GSE103465, and GSE111974 datasets from the Gene Expression Omnibus database were obtained to screen DEGs between RIF and control groups. Gene Ontology analysis, Kyoto Encyclopedia of Genes and Genomes Pathway analysis, Gene Set Enrichment Analysis, and Protein-protein interactions analysis were performed to investigate potential biological functions and signaling pathways. CIBERSORT was used to describe the level of immune infiltration in RIF, and flow cytometry was used to confirm the top two most abundant immune cells detected.

**Results:**

122 downregulated and 66 upregulated DEGs were obtained between RIF and control groups. Six immune-related hub genes were discovered, which were involved in Wnt/-catenin signaling and Notch signaling as a result of our research. The ROC curves revealed that three of the six identified genes (AKT1, PSMB8, and PSMD10) had potential diagnostic values for RIF. Finally, we used cMap analysis to identify potential therapeutic or induced compounds for RIF, among which fulvestrant (estrogen receptor antagonist), bisindolylmaleimide-ix (CDK and PKC inhibitor), and JNK-9L (JNK inhibitor) were thought to influence the pathogenic process of RIF. Furthermore, our findings revealed the level of immune infiltration in RIF by highlighting three signaling pathways (Wnt/-catenin signaling, Notch signaling, and immune response) and three potential diagnostic DEGs (AKT1, PSMB8, and PSMD10).

**Conclusion:**

Importantly, our findings may contribute to the scientific basis for several potential therapeutic agents to improve endometrial receptivity.

## 1 Introduction

Although there is no unified definition for recurrent implantation failure (RIF), it is usually referring to women who fail to achieve a clinical pregnancy after the transfer of at least four high-quality embryos in a minimum of three fresh or frozen cycles in a woman under the age of 40 years (1, 2). As multiple failed cycles can often cause frustration and desperation for the struggling couples, the causes for these failures remain unknown. According to a recently published retrospective cohort study, the incidence of RIF was around 5% in women who had an anatomically normal uterus and underwent consecutive single euploid blastocyst transfer (3), among which 50% of affected cases remain unexplained. It is essential and urgent to determine the etiology of idiopathic RIF and propose novel therapeutic approaches for these patients.

The success of embryo implantation depends on embryonic and endometrial factors. With the innovative breakthrough in preimplantation genetic testing (PGT), abnormal embryos can be discriminated by screening and selection technologies (4). The interventions targeting endometrium have also been more matured in the past decade. For instance, such developments include optimized IVF protocols to synchronize cooperation between the endometrium and the embryo, antibiotic regimens to revert endometrial dysfunction resulting from chronic endometritis (5), and surgical management to rectify congenital uterine malformation and intrauterine adhesion. Nevertheless, endometrium is a complicated tissue composed of various cell types that are dynamically adapted during the window of implantation (WOI) to support the apposition, attachment and invasion of embryo in a synchronic way. The elements involved in this endometrial transformation may open an avenue to elucidate other potential causes and therapeutic targets of implantation failure.

In recent years, increasing accumulated evidence suggests the fundamental role of immunologic factors in embryo implantation. Women with RIF are usually manifested by abnormal immune signaling in the endometrium and peripheral blood, which is particularly characterized by altered Th1/Th2 ratio, and disrupted NK cell and macrophage numbers (6, 7). Additionally, in the endometrium of patients with RIF, the levels of numerous cytokines, chemokines, and their receptors such as IL2, IL6, IFNG, IL17A, IL23A, CCR4, CCR5, CXR3, CCL2, TLR4 are aberrant (8). Further studies are still needed to determine whether these are causative of RIF or just a secondary event. Though the contribution of immune factors in endometrium receptivity can be further explored with the application of RNA-sequencing, the sample sizes in these studies are usually very small, ranging from 9 to 48 samples (9–11), and the immune cell distribution was not analyzed comprehensively. Therefore, it is intriguing to do an in-depth review of the immune network in the endometrium by a systematic analysis of global infiltrating levels of various immune cells and potential immunologic-related markers for the direction of future studies.

Bioinformatics analysis on gene expression microarray has been recommended as a powerful weapon to explore potential biomarkers and signaling pathways in complicated diseases for understanding the pathogenesis and therapeutic development (12). By using microarray data from a publicly accessible database, we further evaluated the differently expressed genes (DEG) in the endometrium of women with RIF and control patients for processing by functional enrichment analysis. To further characterize the features of immune response enriched by aforementioned analysis, we investigated the panorama of immune infiltration by CIBERSORT, and the main findings were preliminarily verified by flow cytometry. With the identification of some immune signaling-related genes, the corresponding ROC curves were plotted for the potential diagnostic values of RIF. Moreover, potential drugs and their potential molecular targets in RIF were identified by cMAP analysis. In this study, we aimed to provide a comprehensive overview of the immune infiltration levels and signaling pathways in women with RIF, and to reveal the potential immune signaling-related biomarkers or therapeutic targets for RIF.

## 2 Materials and methods

### 2.1 RIF datasets

Four independent RIF gene expression profiles (GSE103465, GSE111974, GSE58144, and GSE183837) were downloaded from Gene Expression Omnibus (GEO) database (https://www.ncbi.nlm.nih.gov/geo/). GSE103465, and GSE111974 were exploited as discovery datasets to identify DEGs. GSE58144 and GSE183837 were selected as the validation datasets. GSE103465 is an expression profile based on the GPL16043 platform (GeneChip^®^ PrimeView™ Human Gene Expression Array (with External spike-in RNAs)) and contains samples of fertile control (n=3) and RIF (n=3). GSE111974 was obtained from the microarray platform of Agilent-039494 SurePrint G3 Human GE v2 8x60K Microarray 039381 (Probe Name version), and included 48 endometrium samples (24 control endometrium samples, and 24 RIF endometrium samples). GSE58144 was an expression profile based on the GPL15789 platform (A-UMCU-HS44K-2.0), and contains 71 control endometrium samples, and 43 RIF endometrium samples. GSE183837 was a single-cell RNA-seq (scRNA-seq) dataset of fertile control (n=3) and RIF (n=6), based on the 10X Genomics Chromium platform. The detailed information of these four datasets were listed in **Table 1**.

**Table 1:** Baseline Characteristics.

	GSE103465	GSE111974	GSE58144	GSE183837
Techniques	GPL16043 platform	Agilent-039494 SurePrint G3 Human GE v2 8x60K Microarray 039381	GPL15789 platform	10X Genomics Chromium platform
Sample size	6 (3 control samples, 3 RIF samples)	48 (24 control samples, 24 RIF samples)	114 (71 control samples, 43 RIF samples)	9 (3 control samples, 6 RIF samples)
Age (years) (mean ± SEM)	27.17 ± 3.06	No information	34.37 ± 3.03	33.22 ± 2.28
Timing of biopsy	day LH + 7	No information	day LH + 7	day LH + 7
Inclusion Criteria	Ctrl: had a history of at least one live birth. RIF: had a history of implantation failure from at least three consecutive IVF attempts (including a total of ≥ 4 good-quality embryos).			
Exclusion Criteria	1. tubal obstruction (tubal obstruction factor on hydrosalpinx, salpingitis, etc. were excluded); 2. active pelvic infections, undiagnosed vaginal bleeding, uterine anomalies, endometriosis, karyotype anomalies in one or both partners; 3. unexplained infertility.			

### 2.2 Data preprocessing

The “limma” package (13) in R software (version 4.2.0; https://www.r-project.org/) was used to background correction and quantile normalization of all the raw data files, and the expression values were then obtained. The averages of the probe set of values were calculated as the expression values for the same gene with multiple probe sets (14).

### 2.3 Identification of DEGs

To establish the DEGs between control and RIF samples, the “*limma*” package of R (13) was used. Moreover, a volcano plot was generated to assess the DEGs. |log2 fold change (FC) |≥ 1, adjusted P value < 0.05 were taken as differentially expressed genes between RIF and control endometrium. Volcano map of DEGs was drawn by GraphPad Prism v8.0 software.

### 2.4 Functional enrichment of DEGs

The Gene Ontology (GO) analysis, and Kyoto Encyclopedia of Genes and Genomes (KEGG) pathway analysis were carried out by using R software and the “*clusterProfiler*” package (15). In this analysis symbol codes were converted to Entrez ID using Human genome annotation package “*org.Hs.eg.db*.” The “*ggplot*2” (16), “*pathview*” (17) and “*gplots*” packages of R software to visualize the plots. GSEA software (version 4.1.0, http://www.gsea-msigdb.org/gsea/index.jsp) was used to carry out GSEA analysis (18). The “c2.cp.kegg.v7.5.1.symbols.gmt”, “c5.go.bp.v7.5.1.symbols.gmt”, “c5.go.cc.v7.5.1.symbols.gmt”, “c5.go.mf.v7.5.1.symbols.gmt” was chosen as the reference gene set. Gene set permutations were performed 1,000 times for each analysis. Adjusted P-value < 0.05 and Q value < 0.05 were considered significant for GO and KEGG analysis. The cutoff point of significance was |normalized enrichment score (NES)| > 1, P-value < 0.05, false discovery rate (FDR) Q value < 0.25 for GSEA.

### 2.5 Protein–protein interaction (PPI) network analysis

The STRING database (available online: http://string-db.org) was performed for PPI network prediction. Cytoscape (v.3.7.2) was used for visual representation and further PPI network experimental studies.

### 2.6 Hub gene extraction and submodule analysis

Hub genes play an important role in biological processes. Based on the PPI network, hub genes were screened according to network topology. Cytoscape software (version 3.7.2, cytoHubba and MCODE plug-ins) was used to discover the key targets or subnetworks of complex networks (19, 20). The functional annotation and pathway enrichment analysis of hub genes was used by g:Profiler (https://biit.cs.ut.ee/gprofiler/gost) (21).

### 2.7 Immune infiltration analysis

The different immune cell types of tissues were analyzed by CIBERSORT (22), using normalized gene expression profiles of GSE103465, and GSE111974. LM22, a leukocyte gene signature matrix, contains 547 genes that distinguish 22 human hematopoietic cell phenotypes, including seven T cell types, naïve and memory B cells, plasma cells, NK cells, and myeloid subsets. CIBERSORT results matched ground truth phenotypes in 93% of external datasets of variably purified leukocyte subsets (22). Based on the analysis of LM22, a matrix of 22 kinds of immune cells (naïve B cells, memory B cells, plasma cells, CD8^+^ T cells, CD4^+^ naive T cells, CD4^+^ resting memory T cells, CD4^+^ activated memory T cells, follicular helper T cells, regulatory T cells (Tregs), γδT cells, resting NK cells, activated NK cells, monocytes, M0 macrophages, M1 macrophages, M2 macrophages, resting dendritic cells, activated dendritic cells, resting mast cells, activated mast cells, eosinophils, and neutrophils) was obtained. CIBERSORT P < 0.05 was used to filter the samples, and the percentage of each immune cell type in the samples was calculated and displayed in a bar plot. The “box” package was used to compare the levels of 22 kinds of immune cells between the two groups. The “*ggplot2*” package (16) was adopted to visualize the plots.

### 2.8 ROC analysis

The multivariate modelling with combined selected genes used to identify biomarkers with high sensitivity and specificity for RIF diagnosis by using visualization tool (https://hiplot.com.cn/basic/roc). GSE103465, and GSE111974 datasets were analyzed for training the model, and GSE58144 dataset as validation sample iteratively. The receiver operator characteristic (ROC) curves were plotted and area under curve (AUC) was calculated separately to evaluate the performance of each model using the R packages “*pROC*” (23). And AUC > 0.7 indicated that the model had a good fitting effect.

### 2.9 Screening of potential drugs against RIF

Connectivity map (cMap) online tool (http://clue.io) was used to search for compounds that may cause genomic changes similar or opposite to the changes in RIF patients. Compounds with enrichment score >90 or score <−90 were considered significantly positive or negative compounds, respectively.

### 2.10 Patients’ recruitment for the cohort of validation

The protocol for this study was approved by the Human Research Ethics Committees of Obstetrics and Gynecology Hospital of Fudan University, and written informed consent was obtained from all participants. Human endometrial tissues were collected from women attending the Department of Assisted Reproduction, a dedicated research clinic at the Obstetrics & Gynecology Hospital of Fudan University. Surplus tissue from endometrial biopsies obtained for diagnostic purposes at the Department of Assisted Reproduction was used for this study. The timing of endometrial biopsy was 5 days after ovulation evaluated by ultrasonography, equivalent to LH+7 (hallmark of WOI) in a natural cycle. The definition of RIF was consistent with the studies recruited for bioinformatics. The control group recruited women who had a history of pregnancy but received the assistance of reproductive technology due to obstruction of fallopian tube or male infertility. All the samples were transported to the laboratory on ice in Dulbecco’s modified Eagle’s medium (DMEM)/F-12 (Gibco) for further study.

### 2.11 Sample preparation and immunocyte isolation

The procedures of sample preparation and immune cell isolation were described with details in our previous studies (24). In brief, the endometrial tissue samples were minced into 1 mm^3^ pieces on ice and subsequently digested with 1 mg/mL collagenase type IV (Sigma, USA). The tissue pieces were later filtered through a 70 μm cell strainer (Falcon, USA), followed by centrifugation at 400 g for 8 min for the collection of all types of cells. Later, the cells were resuspended in DMEM/F-12 containing 10% fetal bovine serum (FBS; Hyclone, Logan, UT, USA), plated on culture flasks and incubated in a humidified incubator with 5% CO_2_ at 37 °C. Finally, after 24 hours, the supernatant was removed and centrifuged at 400 g at 4 °C for flow cytometry analysis.

### 2.12 Flow cytometry analysis (FCM)

Flow cytometry analysis (FCM) was used to validate the main changes in endometrial immunocytes between women with RIF and controls. All antibodies were from Biolegend (San Diego, CA, USA). The antibodies to detect endometrial NK cells and macrophages included: APC/Cyanine 7 (APC/Cy7)-conjugated anti-human CD45, fluorescein isothiocyanate (FITC)-conjugated anti-human CD3, brilliant violent 605-conjugated anti-human CD56, PE-Cy7-conjugated anti-human CD14, and PE-conjugated anti-human CD16. Staining was performed with the above antibodies (5 μL separately) at room temperature for 30 minutes in the dark. In addition, an isotype IgG antibody of each marker (5 μL separately) was used as the control. Human Trustain FcX (cat. No. 422301; Biolegend, Inc.) was used to block Fc receptors prior to flow cytometry. Subsequently, cells were washed twice and resuspended in PBS for flow cytometry analysis. Samples were analyzed using a CytoFLEX flow cytometer (Beckman Coulter, Inc.), and data were analyzed using FlowJo (version 10.07, FlowJo LLC.).

### 2.13 Statistical analysis

Statistical analysis was performed using SPSS (version 23.0, Chicago, IL) and GraphPad Prism (version 8, San Diego, CA) software. Continuous variables were expressed as mean ± standard deviation (SD), and differences between the two groups were compared using Student’s t-test for normally distributed variables. A Kruskal–Wallis H test with Dunn’s multiple-comparisons test was used for continuous variables with a non-normal distribution, and the results were presented as the median and interquartile range. The sensitivity and specificity of feature genes to distinguish RIF from controls were assessed using a receiver operating characteristic (ROC) curve. Differences were considered statistically significant when P < 0.05.

## 3 Results

### 3.1 Data preprocessing and heat map of top 50 genes in RIF and Ctrl groups

**Figure 1:**
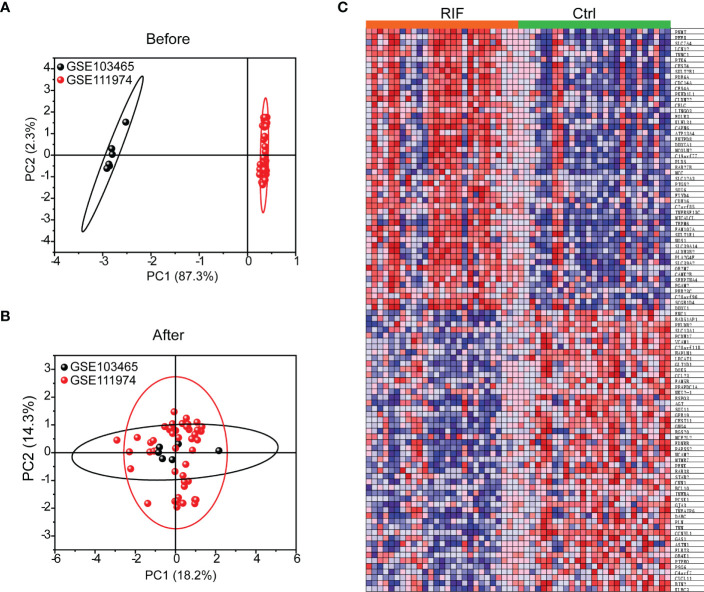
Data Preprocessing and Heat map of top 50 genes in RIF and Ctrl groups. **(A, B)** PCA analysis of GSE103465, and GSE111974 expression profiles before **(A)** and after **(B)** normalization. **(C)** Expression of top 50 genes in RIF and Ctrl groups was presented by heatmap.

All expression values of GSE103465 and GSE111974 datasets were normalized by principal component analysis (PCA), a method to identify strong patterns in large, complex datasets, which is widely used in data reduction technique captures the essential information in high-dimensional data by identifying principal components that account for most of the variability in the dataset. The data before and after normalization are presented in **Figures 1A, B**. The heatmap of the top 50 DEGs between RIF and Ctrl groups is shown in **Figure 1C**.

### 3.2 Identification, PPI network and module analysis of DEGs

**Figure 2:**
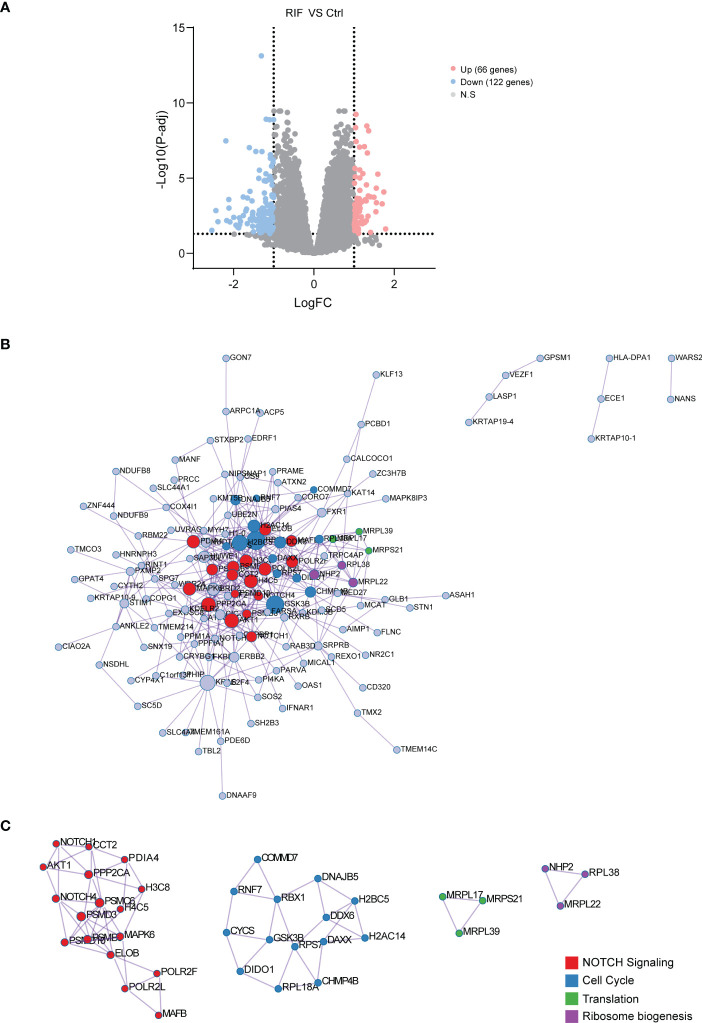
Identification, PPI Network and Module Analysis of DEGs. **(A)** The 188 DEGs were visualized by volcano map. **(B)** PPI network constructed with the DEGs from GSE103465, and GSE111974 datasets. **(C)** The significant module identified from the PPI network using the molecular complex detection (MCODE) method with a score of ≥ 5.0. Different nodes’ color present different functions.

The two discovered datasets contained 18301 genes with 27 RIF and 27 Ctrl samples. In total, 188 DEGs (122 genes were downregulated and 66 genes were upregulated) were identified (**Figure 2A** and **Table 2**). With PPI enrichment analysis being performed, significant modules were identified (**Figures 2B, C**). The four MCODE components extracted were found to be mainly associated with NOTCH signaling pathway, cell cycle, translation and ribosome biogenesis.

**Table 2:** Up and Down DEGs.

Expression	Gene Name
Up	C1orf131, CALCOCO1, STIM1, PSMD3, RNF7, COMMD7, CD320, TMEM214, KIAA0415, RBX1, KRTAP20-1, MRPL22, STXBP2, C2orf44, RAB3D, MANF, MRPL17, SLC44A1, AMT, CHMP4B, MICAL1, KRTAP10-1, RPL18A, HNRNPH3, SNX19, RBM22, TOR3A, BRD2, AGPAT6, GSK3B, C14orf142, SPG7, ARPC1A, ILF2, KRTAP10-9, MED27, SCD5, PXMP2, DHTKD1, NMB, SLCO2A1, H1F0, CYCS, GPSM1, PCBD1, EGFL6, NDUFB8, ERBB2, HIST1H2BI, GPR32, FKBP3, RPS7, HLA-DPA1, KLF13, KDM5B, NHP2, DDX6, PSMD10, TMCO3, COX4I1, DIDO1, NANS, CORO7, FXR1, NIPSNAP1, KCNMA1
Down	FAM96A, HIST1H2BF, ECE1, PSMC6, RXRB, CLDN3, SPEF2, MRPS21, PDE6D, DAXX, UBE2N, TRPC4AP, C9orf23, CYP4X1, CYTH2, PPP2CA, E2F4, CCNB1IP1, NUDT21, MUC16, AIM1, GLTP, NOTCH1, COPG, PDE6G, HUWE1, RNASE8, CRISP2, KDELR2, SUV420H1, IFNAR1, MAFG, EMP1, HIST1H2AJ, TMEM161A, ACP5, MCAT, PIAS4, NSDHL, TMEM14C, KRAS, PHIP, AKT1, UVRAG, CIC, GTPBP1, TRIM9, PARVA, PPFIA1, ZC3H7B, HIST1H4E, ARRDC2, C19orf56, LASP1, ASAH1, TSPAN6, POLR2L, WARS2, GLB1, PDIA4, PSMB8, PPM1A, MAPK8IP3, SRPRB, SC5DL, SEC14L1, NR2C1, ARHGAP15, MRPL39, ATP6V1C1, OAS1, PRAME, POLR2F, ATP13A5, MAPK6, FARSA, HIST1H3G, TSPAN14, HERC6, NOTCH3, WDR24, MYH7, CCT2, MAFB, NOTCH4, TMX2, AIMP1, OS9, REXO1, ZNF444, TMEM211, C10orf137, OBFC1, SLC4A4, FLNC, VEZF1, TCEB2, ANKLE2, FILIP1L, NDUFB9, RPL38, C10orf26, ABP1, SH2B3, TBL2, PLAC9, PRCC, SOS2, C20orf194, TMEM60, RNASE6, NELL2, SAP30L, ATXN2, RINT1, PI4KA, FCGBP, DNAJB5, KRTAP19-4, CSRP2BP, EXOSC8, RHBDF1

### 3.3 Functional enrichment analysis of DEGs

**Figure 3:**
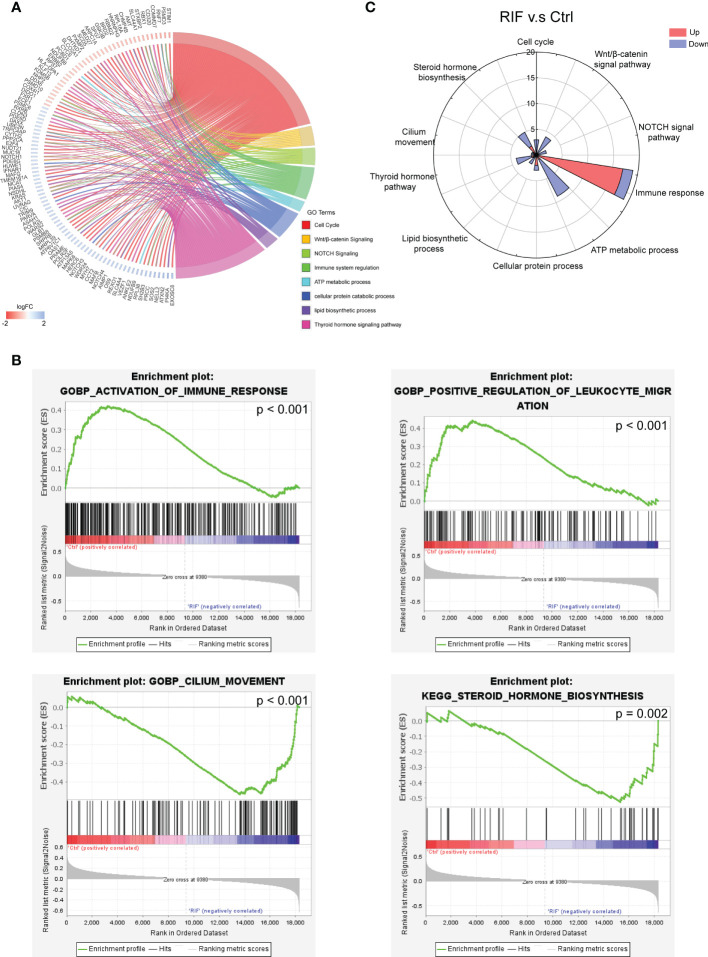
Functional Enrichment Analysis of DEGs. **(A, B)** GO analysis, KEGG pathway analysis **(A)**, and GSEA enrichment analysis **(B)** of DEGs. **(C)** Up or down gene numbers related to cell cycle, signaling by Wnt/β-catenin, signaling by NOTCH, immune system regulation, ATP metabolic process, cellular protein catabolic process, lipid biosynthetic process, and thyroid hormone signaling pathway in RIF group from validation dataset (GSE183837) were presented by rose diagram.

To further investigate the biological functions of the 188 DEGs, GO analysis, KEGG pathway analysis and GSEA were performed. GO analysis and KEGG pathway analysis demonstrated that DEGs were mainly associated with cell cycle, Wnt/β-catenin signaling, NOTCH signaling, immune system regulation, ATP metabolic process, cellular protein catabolic process, lipid biosynthetic process, and thyroid hormone signaling pathway (**Figure 3A**). While for the GSEA analysis, activation of immune response and positive regulation of leukocyte migration were remarkably increased in patients with RIF, and cilium movement and steroid hormone biosynthesis were suppressed in patients with RIF (**Figure 3B**). Besides immune system regulation, rose diagram of the validation dataset (GSE183837) showed the genes in the pathways of cell cycle, the Wnt/β-catenin signaling, NOTCH signaling, ATP metabolic process, cellular protein catabolic process, lipid biosynthetic process, and thyroid hormone signaling pathway were significantly down-regulated in patients with RIF (**Figure 3C**).

### 3.4 Identification and functional enrichment analysis of hub genes

**Figure 4:**
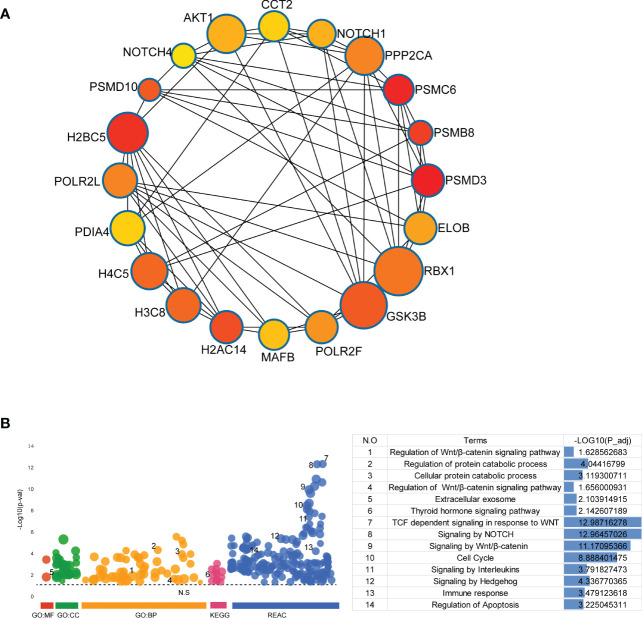
Identification and Functional Enrichment Analysis of Hub Genes. **(A)** The top 20 hub genes were discovered by Cytoscape software (version 3.7.2, cytoHubba plug-ins). **(B)** GO analysis, KEGG pathway analysis and REAC analysis of hub genes were performed by g:Profiler (https://biit.cs.ut.ee/gprofiler/gost).

To further evaluate the significance of the genes in the PPI network of DEGs, the top 20 hub genes were obtained from PPI network by using cytoHubba and presented in **Figure 4A**. GO analysis, KEGG pathway analysis and REAC analysis demonstrated that hub genes were mainly associated with cell cycle, the Wnt/β-catenin signaling, NOTCH signaling, immune response, cellular protein catabolic process, and thyroid hormone signaling pathway (**Figure 4B**).

### 3.5 Immune infiltration analysis

**Figure 5:**
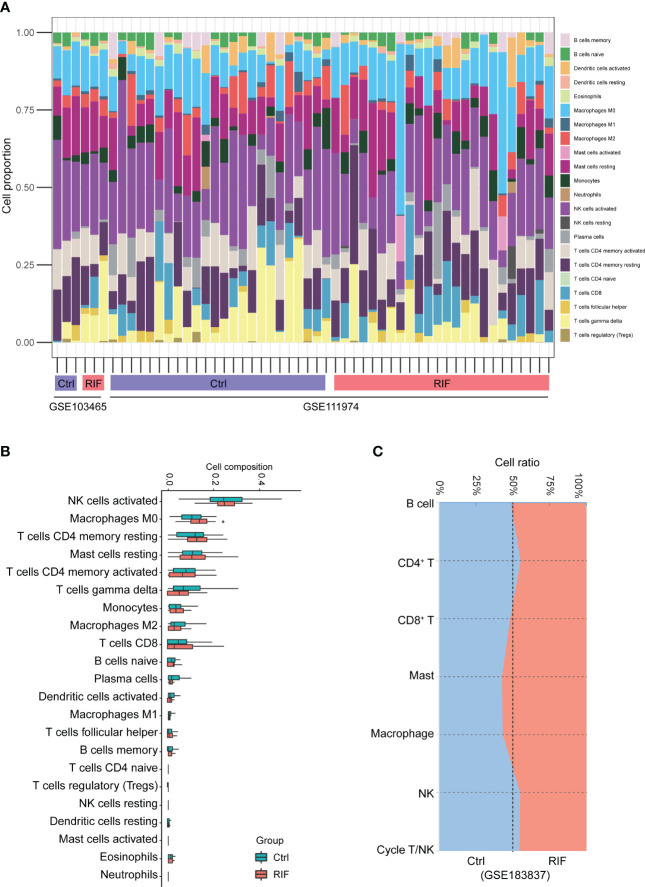
The landscape of immune infiltration between RIF and control samples. **(A)** The relative percentage of 22 types of immune cells. **(B)** The difference of immune infiltration between RIF and controls. The control group was marked as blue color and RIF group was marked as red color. **(C)** Stacked area plot showed the relative percentage of 7 types of immune cells from validation dataset (GSE183837). Data are presented as the median and the interquartile range. Statistical significance (Kruskal–Wallis test with Dunn’s multiple-comparison test): *p < 0.05.

**Figure 6:**
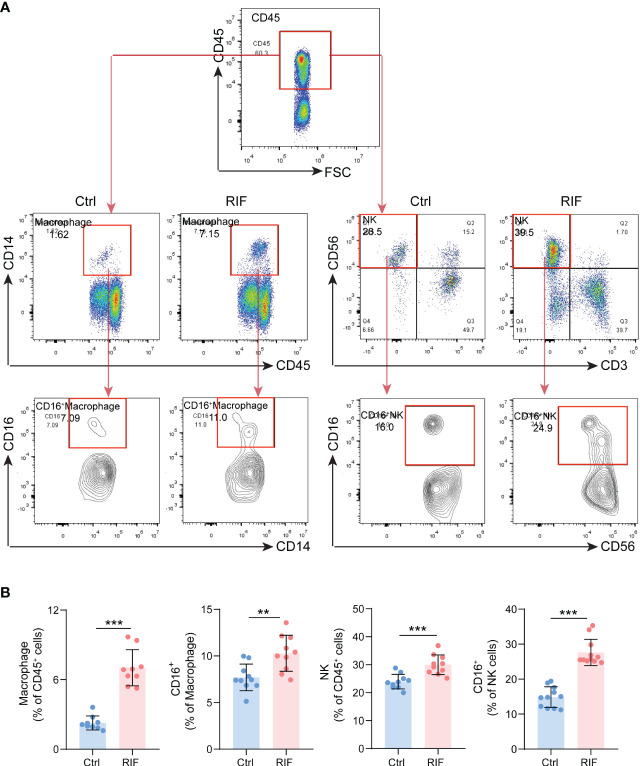
The Proportions of Macrophage and NK Cells. **(A)** The gating strategy for identifying macrophage and NK cells from endometrial leukocytes. **(B)** The quantitative analysis of the proportions of macrophage, NK cells, CD16^+^ macrophage and CD16^+^ NK cells (n=10) in endometrium from control and RIF patients. Data are presented as the mean ± standard error of the mean. Statistical significance (Student’s t-test): **p < 0.01, ***p < 0.001.

More importantly, functional enrichment analysis of DEGs exhibited a significant enrichment of immune response in endometrium from RIF patients. Herein, we further performed the CIBERSORT algorithm to compare the immune cells infiltration level between patients with RIF and the control group (**Figure 5A**). The percentage of each of the 22 types of immune cells in each sample is shown in the bar plot, and it was intuitively illustrated that most of the endometrial immune cells were NK cells, followed by macrophages, accounting for approximately 30% of total cells which is similar to previous studies (25, 26). The boxplot of the infiltration levels of various immune cells was sorted according to their median proportion in the RIF group, suggesting that NK and macrophages were the top two of the most abundant immune cells. The relative proportions of activated NK cells and macrophages were not significantly different between the two groups, but M0 macrophages were significantly increased in women with RIF (p<0.05) (**Figure 5B**). These findings were validated by another set of data (GSE183837). As shown in **Figure 5C**, we found that the proportion of endometrial immune cells in patients with RIF and Ctrl patients also changed by single-cell RNA sequencing (GSE183837). Since NK cells and macrophages were the top two abundant cells in endometrium during the WOI and their crucial roles in implantation is unneglectable, we further measured the proportions of endometrial NK cells and macrophages of patients with RIF and healthy women by FCM to validate the changes. Our data showed that the proportions of macrophages and NK cells were significantly increased in the endometrium of patients with RIF, and the proportions of CD16^+^ macrophage and CD16^+^ NK cells were also elevated (**Figures 6A, B**), which echoed the results of array in **Figure 5** partly. These data implied that RIF was associated with abnormally elevated NK cells and macrophages numbers, as well as increased cytotoxicity.

### 3.6 Expression levels and diagnostic significance of hub genes

**Figure 7:**
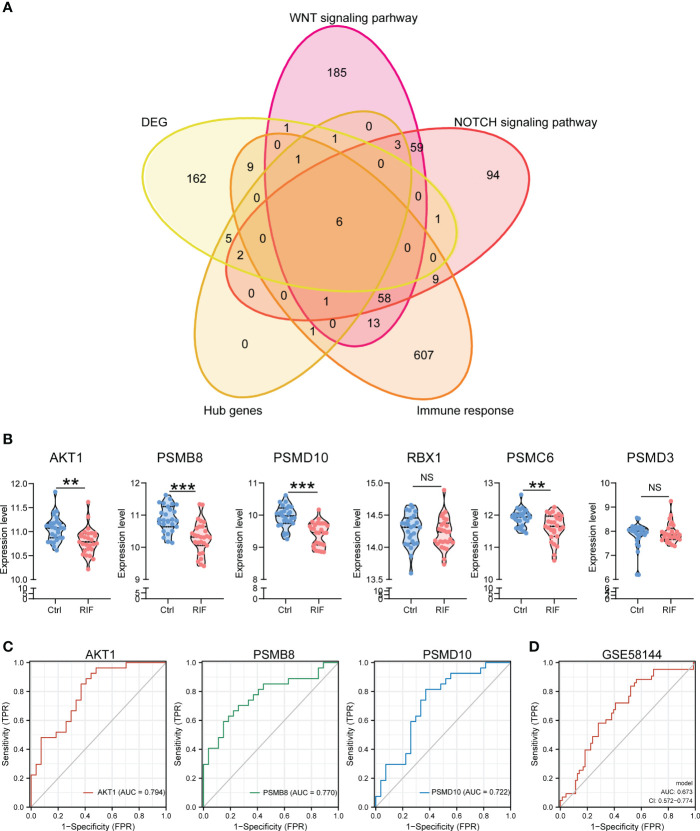
Expression Levels and Diagnose Significance of Hub Genes. **(A)** Venn diagram of common hub genes identified among DEGs, Wnt/β-catenin signaling pathway, NOTCH signaling pathway, immune response, and hub genes. **(B)** The expression levels of 6 hub genes in GSE103465, and GSE111974 datasets. **(C)** The GSE103465, and GSE111974 datasets were used to train the diagnostic effectiveness of the biomarkers for RIF by ROC analysis. **(D)** The GSE58144 dataset was used to validate the diagnostic effectiveness of the biomarkers (AKT1, PSMB8, and PSMD10) for RIF by ROC analysis. Data are presented as the median and the interquartile range. Statistical significance (Kruskal–Wallis test with Dunn’s multiple-comparison test): NS, no significant difference, **p < 0.01, ***p < 0.001.

Regarding to the importance of Wnt/β-catenin signaling pathway, NOTCH signaling pathway, and immune response in RIF, we identified 6 genes (AKT1, PSMB8, PSMD10, RBX1, PSMC6, and PSMD3) from the hub genes (**Figure 7A**). As they were all related to these key pathways, 4 of them were remarkably decreased in the endometrium of patients with RIF according to the GSE103465 and GSE111974 datasets (**Figure 7B**). To determine which hub genes might act as diagnostic markers for patients with RIF, ROC analysis was conducted to explore the diagnostic values of the 6 hub genes for RIF. The results showed that AKT1, PSMB8, and PSMD10 had potential predictive values for RIF in the model of training sets (AUC > 0.7) (**Figure 7C**). We then validated the model composed of the above 3 qualified genes (AKT1, PSMB8, and PSMD10) in the validation set, the AUC was found to be 0.673 (**Figure 7D**).

### 3.7 Drug-target genes interaction network analysis for RIF

**Figure 8:**
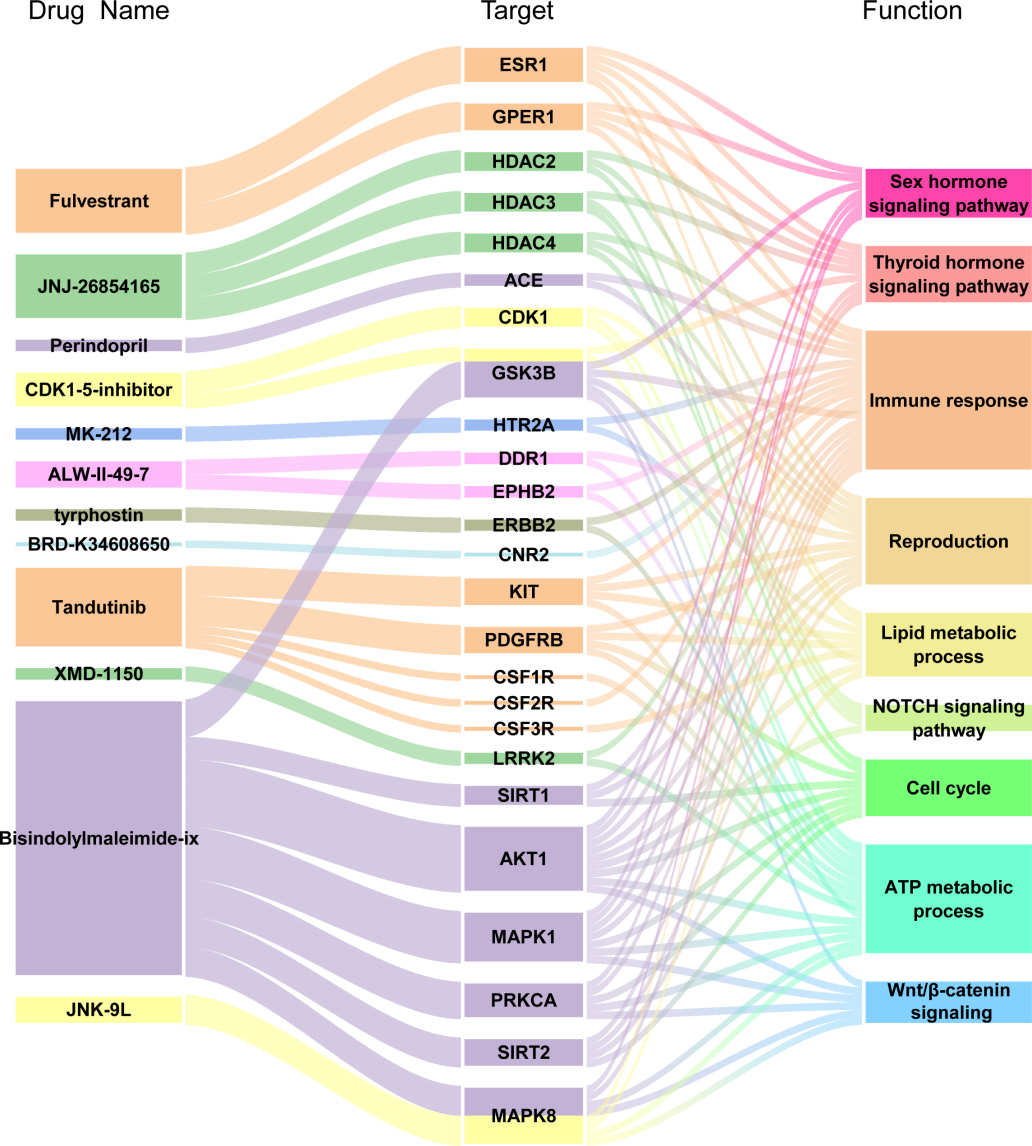
Drug-Target genes Interaction Network Analysis. From the left to right of alluvial diagram: the potential pharmaceuticals, target genes and enriched pathways by GO and KEGG analysis.

To explore potential therapeutic or induced compounds for RIF, we queried the cMap touchstone database with expression signatures from DEGs. Compounds with enrichment score >90 or <−90, which were regarded as significantly positive or negative compounds, respectively, and their target genes were shown together (**Figure 8** and **Table 3**). Among the drugs with the highest connectivity scores, fulvestrant (estrogen receptor antagonist) was found to be positively correlated with the occurrence of RIF. Among the drugs with the lowest connectivity scores, bisindolylmaleimide-ix (CDK and PKC inhibitor) and JNK-9L (JNK inhibitor) were shown to be negatively correlated with the occurrence of RIF, indicating their potential as therapeutic compounds. These compounds could influence the pathophysiology of RIF by regulating relevant pathways, such as cell cycle, the Wnt/β-catenin signaling, NOTCH signaling, immune response, reproduction, and thyroid hormone signaling pathway. Notably, AKT1, on the hub genes list, was one of bisindolylmaleimide-ix’s targets.

**Table 3:** Information of Top 8 prospective drugs with significant scores for the treatment.

Score	Name	Target	MOA
97.58	fulvestrant	ESR1, EPHX2, ESR2, GPER1	Estrogen receptor antagonist
96.03	JNJ-26854165	HDAC1, HDAC10, HDAC11, HDAC2, HDAC3, HDAC4, HDAC5, HDAC6, HDAC7, HDAC8, HDAC9, MDM2	HDAC inhibitor
95.94	huperzine-a	ACHE	Acetylcholinesterase inhibitor
94.86	perindopril	ACE	ACE inhibitor
94.26	CDK1-5-inhibitor	CDK1, CDK5, GSK3B	CDK inhibitor, Glycogen synthase kinase inhibitor
93.87	SU-11274	MET	Hepatocyte growth factor receptor inhibitor, Tyrosine kinase inhibitor
93.87	MK-212	HTR2A, HTR2B, HTR2C	Serotonin receptor agonist
93.5	CO-101244	GRIN2B	Ionotropic glutamate receptor antagonist
93.03	ALW-II-49-7	DDR1, EPHA2, EPHA4, EPHA7, EPHB2, EPHB4, EPHB6	Ephrin inhibitor
93.01	tyrphostin	ERBB2	EGFR inhibitor
92.25	BRD-K34608650	CNR2	Cannabinoid receptor agonist
90.54	hymecromone	MAOA	Monoamine oxidase inhibitor
90.34	tandutinib	FLT3, KIT, PDGFRA, PDGFRB, CSF1R, PDGFD	FLT3 inhibitor, KIT inhibitor, PDGFR receptor inhibitor
-90.43	XMD-1150	LRRK2	Leucine rich repeat kinase inhibitor
-91.23	bisindolylmaleimide-ix	SIRT1, AKT1, GSK3B, LCK, LRRK2, MAPK1, MAPK11, MAPK12, MAPK14, MAPK8, PRKCA, ROCK1, RPS6KB1, SIRT2	CDK inhibitor, PKC inhibitor
-95.03	JNK-9L	MAPK8	JNK inhibitor
-95.65	Ala-Ala-Phe-CMK	TPP2	Tripeptidyl peptidase inhibitor

## 4 Discussion

It is a widely-accepted consensus that the process of embryo implantation is analogous to seed germination, in which the quality of the seed (the embryo) and the receptivity of soil (the endometrium) are both indispensable to successful implantation. In order to promote the success for embryo implantation, the endometrium undergoes a series of changes in functioning signaling pathways and immune cell distribution, subsequently resulting in the maternal immunonutrition and tolerance of the semiallogenic fetus. To comprehend the molecular pathways connected with endometrial receptivity and the foundations of RIF, it is essential to investigate the disruptions in signaling pathways and immune responses in the local endometrium milieu.

The exact mechanisms underlying endometrial immunomodulation by which the embryo is shielded from the maternal immune attack remained poorly understood. As one of the predominant leukocytes in the endometrium, the effects of uNK cells on embryo implantation should not be overlooked (27). In the non-pregnant endometrium, uNK cells are mostly quiescent but they are capable to differentiate throughout the menstrual cycle in preparation for pregnancy (28). When embryo implantation is accomplished, uNK cells contribute to placentation by encouraging trophoblast invasion and spiral artery remodeling to ensure sufficient maternal-fetal material exchange (29). Aberrant uNK activity may produce undesirable outcomes during trophoblast invasion, such as vascular remodeling, local ischemia, and oxidative stress, which are detrimental to implantation (30). According to a latest systematic review and meta-analysis (27), the proportion of CD56^+^ uNK was significantly elevated in patients with RIF when compared with healthy controls. Moreover, most studies found lower expressions of inhibitory receptors and increased expressions of angiogenic factors in uNK cells of women with RIF. These studies may indicate that the assessment of uNK level/activity may be developed as diagnostic tool for RIF. However, a standard reference range would still need to be established prior for use in the clinical setting. In addition to NK cells, macrophages are another significant regulator of the embryo implantation microenvironment. They have been shown to play an important role in implantation, placentation, and embryo development (6). These cells may facilitate trophoblast invasion into spiral arteries, which is related to vascular remodeling at the maternal-fetal interface. Ideal pregnancy outcome calls for appropriate proportion of M1/M2 macrophage ratio as well. However, once distorted, several complications during pregnancy have been shown to occur, including preeclampsia, intrauterine growth restriction, and RIF (31). In line with previous studies, our data validated the higher proportions of uNK cells and macrophages in RIF, notably CD16^+^ NK cells and CD16^+^ macrophages, which revealed stronger cytotoxicity to likely result in maternal immune rejection of the fetus. Nevertheless, further experimental research is still warranted for elucidating the underlying pathophysiology of NK cells and macrophages alterations.

As depicted above, we emphasized the Wnt/β-catenin signaling as a RIF-related signaling pathway by a series of functional enrichment analysis. As shown in previous literature, Wnt/β-catenin signaling acts as a vital part involved in various biological processes ranging from development to early animal evolution and cancer (32). In particular, female reproduction in mammals was shown to be regulated by Wnt4 signaling (33). It has been reported in mice that uterine Wnt/β-catenin signaling is essential for implantation, and the suppression of Wnt4/β-catenin signaling can interferes with the process of embryo implantation (34). On the basis of animal studies, either depletion or overexpression of Wnt/β-catenin in the endometrium would led to implantation failure in mice (35). Meanwhile, Franco et al. demonstrated that the conditional knockout of Wnt4 in the uterus results in infertility and abnormal decidualization in mice (36). Similarly, Zhou et al. discovered during decidualization that EHD1 inhibited the Wnt4/-catenin pathway in patients with RIF by regulating LRP5/6 protein activity *via* the endocytic route. As a Wnt4 agonist could restore a defective decidualization process, this suggesting that modulation of the EHD1-Wnt4 pathway may be a viable therapeutic target for enhancing endometrial receptivity in women with RIF (37). While studies in human, clinical research has found that the expression of seven Wnt genes (Wnt 2, 3, 4, 5a, 7a, 7b and 10b) in the endometrium during pregnancy occupied a decisive position in the regulation of survival, proliferation and differentiation in endometrial stromal cell (38).

Another highlighted pathway in this current study was the Notch signaling pathway. As an evolutionarily conserved pathway, Notch signaling has been shown to regulate cell proliferation, invasion, differentiation, and apoptosis (39), which contributed significantly in endometrial remodeling. Additionally, during implantation and placentation, Notch signaling is part and parcel of maternal-fetal communication (40). As reported in previous literature, aberrant activation of canonical Notch1 signaling in the mouse uterus could decrease the level of progesterone receptor by hypermethylation that can lead to infertility (41). Likewise, hampered Notch signaling in mice is linked to the development of endometriosis which contributes to dysdecidualization through the down-regulation of FOXO1, which might lead to subsequent implantation failure (42). In a recent study involving women with endometrial disorders including RIF, endometriosis, and PCOS, the expression pattern of NOTCH pathway molecules including NOTCH1, 3, JAG1, 2, and survivin in the mid-luteal phase were distinctly expressed in the patient groups compared to controls (43).

These two pathways have also been demonstrated to regulate immune cell differentiation and activation in autoimmune diseases and cancers, which are commonly proposed to be a potential therapeutic target for immunotherapies (44, 45). Whether the downregulation in Wnt/β-catenin signaling and Notch signaling in women with RIF can cause disrupted endometrial immune cell proportions or not, it is still a promising direction to further dissect the underlying mechanisms of RIF. In this regard, Nevertheless, we identified 3 hug genes (AKT1, PSMB8 and PSMD10) that have strong correlation to the two signaling pathways and immune response when discriminating women with RIF from controls. However, the performance of the predictive model was found to be unsatisfactory. This might be due to the likelihood of small sample size. Hence, further studies with a larger sample size are still needed to explore the potential of these hug genes for elucidating their key pathological pathways of RIF for potential clinical applications. In addition to diagnostic values, compounds (fulvestrant, bisindolylmaleimide-ix and JNK-9L) with potential to interfere the pathogenesis of RIF were identified based on the DEGs between women with RIF and controls. However, the exact effects of these compounds on endometrium and embryo implantation were not validated with additional studies. Further investigations on this aspect are still warranted.

**Figure 9:**
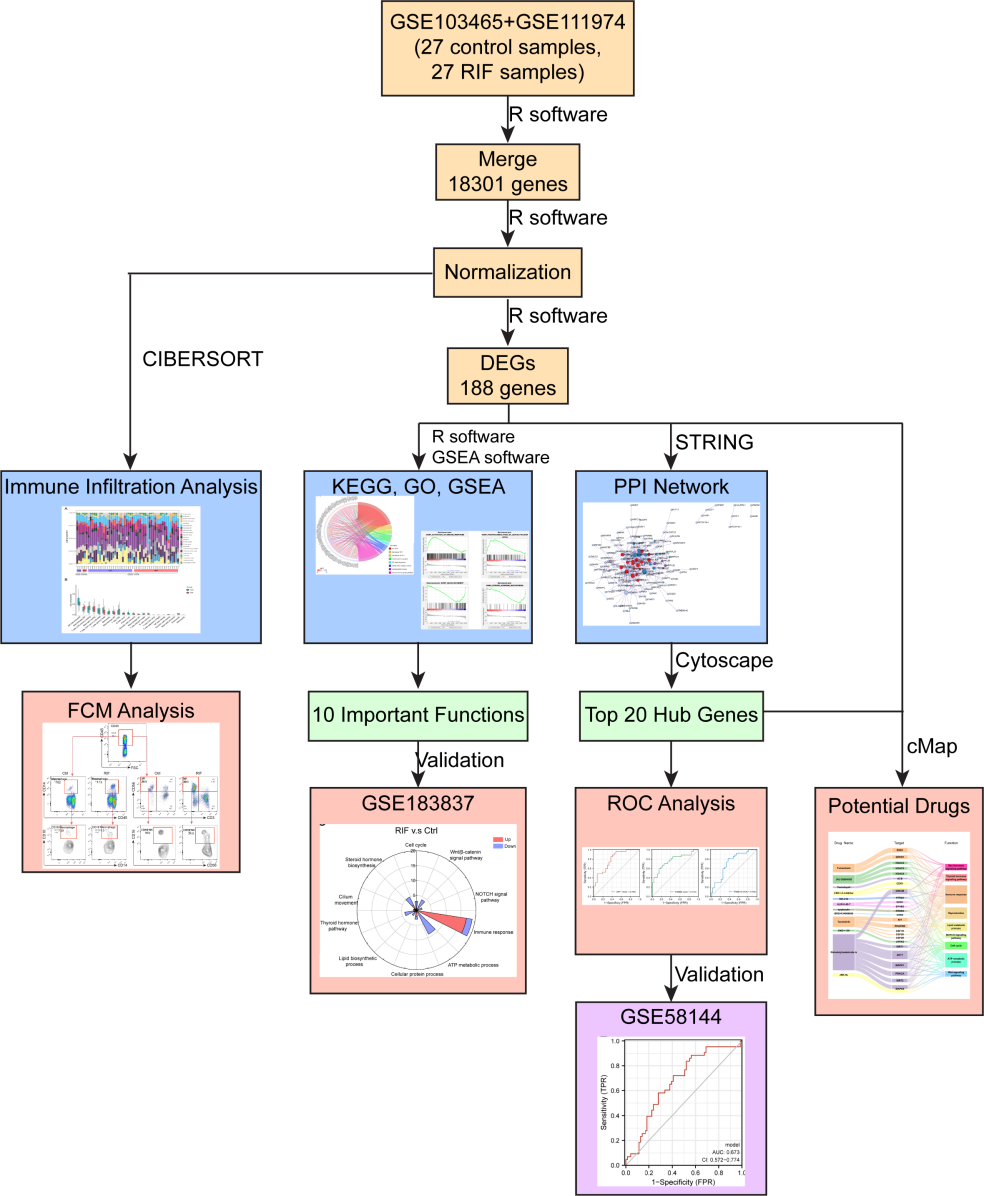
Diagram of the study design. Discover datasets, GSE103465, and GSE111974, contained 18301 genes with 27 RIF and 27 Ctrl samples. In total, 188 DEGs were identified: 122 genes were downregulated and 66 genes were upregulated. Ten important pathways related to RIF were enriched, and were validated by validation dataset (GSE183837). Top 20 hub genes were identified by Cytoscape through PPI network, and three of them may have potential predictive values for RIF in the validation set (GSE58144). Endometrial macrophages’ infiltration level was increased in patients with RIF, compared to the control group, which were performed by CIBERSORT algorithm, and validated by FCM analysis. Bisindolylmaleimide-ix and JNK-9L may be potential therapeutic compounds for RIF, related to the result of cMap analysis.

In summary, our study demonstrated a significant difference in the transcriptional profiles between RIF group and the control group (**Figure 9**). Based on the 122 downregulated and 66 upregulated DEGs, PPI network and module analysis and functional enrichment analysis were performed. Hub genes were further identified by STRING and CytoHubba. Upon our analysis, Wnt/β-catenin signaling pathways, NOTCH signaling pathways, and immune response were the top 3 enriched pathways involved in RIF. With our in-depth analysis, we calculated the immune infiltration level of 22 types of immune cells on validating the increase of NK cells and macrophages in women with RIF. Moreover, the DEGs were assessed to identify potential diagnostic markers and therapeutic compounds for RIF in providing strategies to tackle RIF. However, studies using a larger sample size are still needed to validate the results of the potential genes to potentially be used for diagnosis or prediction of RIF. Another limitation of this study is the lack of functional studies for the potential therapeutic compounds. Nevertheless, our current findings have helped to shed lights for potential directions toward understanding the importance of immunity during RIF.

## Data availability statement

Publicly available datasets were analyzed in this study. This data can be found here: https://www.ncbi.nlm.nih.gov/geo/; GSE103465, GSE111974, GSE58144, and GSE183837.

## Ethics statement

The studies involving human participants were reviewed and approved by Human Research Ethics Committees of Obstetrics and Gynecology Hospital of Fudan University. The patients/participants provided their written informed consent to participate in this study.

## Author contributions

Z-ZL and JZ conducted all experiments, prepared the figures and the manuscript, and were responsible for bioinformatics analysis. W-JZ, J-WS, H-LY, S-LY and FX helped sample collection. J-NW helped data analysis. M-QL and TZ designed, initiated and supervised the project and edited the manuscript. All authors contributed to the article and approved the submitted version.
